# The Gene Expression of Proteins Involved in Intercellular Signaling and Neurodegeneration in the Substantia Nigra in a Mouse Subchronic Model of Parkinson’s Disease

**DOI:** 10.3390/ijms24033027

**Published:** 2023-02-03

**Authors:** Anna Kolacheva, Ekaterina Pavlova, Alyona Bannikova, Vsevolod Bogdanov, Dmitry Troshev, Michael Ugrumov

**Affiliations:** Koltzov Institute of Developmental Biology, Russian Academy of Sciences, 26 Vavilova Street, 119334 Moscow, Russia

**Keywords:** Parkinson’s disease, modeling of Parkinson’s disease, dopaminergic neurons, substantia nigra, neurodegeneration, gene expression, PCR, mice

## Abstract

Given the limited access to clinical material for studying the pathogenesis of Parkinson’s disease (PD), these studies should be carried out on experimental models. We have recently developed a subchronic model of the progressive development of PD with a gradual transition from the preclinical (asymptomatic) stage to the clinical (symptomatic) one. The aim of this study was to evaluate changes in the expression of a wide range of genes in the substantia nigra (SN), the central link in the regulation of motor function, in mice in our subchronic model of PD. We have found changes in the expression of a number of genes encoding enzymes involved in the synthesis and degradation of dopamine as well as proteins involved in the vesicular cycle, axonal transport, protein degradation in the proteasome system, neuroinflammation, and cell death in the SN of our mouse model of the clinical stage of PD. Similar changes in gene expression were previously demonstrated in patients (postmortem), indicating good reproducibility of PD in our model. Further analysis of the gene expression in the SN of mice has shown that the expression of some genes also changes in the model of the preclinical stage, when dopaminergic neurons have not yet died. Thus, this study opens up broad prospects for further evaluation of the molecular mechanisms of PD pathogenesis and the development of a test system for drug screening.

## 1. Introduction

Parkinson’s disease (PD) is one of the most common and severe socially significant neurodegenerative diseases affecting more than 16 million people worldwide [1,2]. A key element in the pathogenesis of PD is the death of dopaminergic (DAergic) neurons of the nigrostriatal system of the brain, which are involved in the regulation of motor function. PD develops for a long time (5–20 years) without the manifestation of motor symptoms, despite the progressive death of DAergic neurons [3]. Motor symptoms, which are used to diagnose PD, appear after the death of 40–60% of DAergic neurons in the substantia nigra (SN) and a 70–80% decrease in the concentration of dopamine (DA) in the striatum. Symptomatic treatment that begins at this time is aimed at compensating for the DA deficiency. However, it does not protect patients from disability and death.

Based on the foregoing, one of the most important priorities of neurology is the development of both early (preclinical) diagnosis of PD, long before the onset of motor symptoms, and preventive neuroprotective treatment aimed at stopping or at least slowing down neurodegeneration. It is believed that such treatment will significantly prolong the preclinical (asymptomatic) stage of PD and, hence, the period of a patient’s comfortable life while maintaining normal social and physical activity. Neuroprotective therapy could also be used at the clinical stage of PD in combination with symptomatic therapy [4].

Successful development of neuroprotective therapy requires deep knowledge of the cellular and molecular mechanisms of neurodegeneration that dominate the preclinical and clinical stages of PD [5]. Conducting such studies on patients at the clinical stage of PD is extremely difficult, and at the preclinical stage, it is impossible due to the lack of early diagnosis. Therefore, such studies should be carried out mainly on experimental models that reproduce the progressive development of PD, including a continuous transition from the preclinical stage to the clinical stage.

The aim of this study was to evaluate the gene expression of proteins involved in DA metabolism and proteins involved in the molecular mechanisms of neurodegeneration in the SN of mice in a recently developed subchronic model of the preclinical and clinical stages of PD using 1-methyl-4-phenyl-1,2,3,6-tetrahydropyridine (MPTP) [6]. Particular attention was given to the expression of genes encoding proteins such as enzymes for the synthesis and degradation of DA, axonal transport, the proteasome system, neuroinflammation, and cell death. Subsequent comparison of the expression of these genes in mice in our subchronic models of the preclinical and clinical stages of PD suggests which proteins play an important role in the molecular mechanisms of the progressive development of PD.

## 2. Results

**Figure 1:**
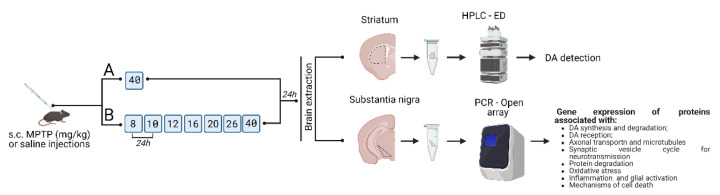
Design of experiments with subcutaneous administration of 1-methyl-4-phenyl-1,2,3,6-tetrahydropyridine (MPTP) to mice: (A) twice at doses of 8 and 10 mg/kg; (B) seven times at gradually increasing doses from 8 to 40 mg/kg, followed by obtaining materials 24 h after the last injection for the analysis of dopamine levels in the striatum and expression of genes of interest in the substantia nigra. This figure was created using BioRender. DA, dopamine; MPTP, 1–methyl–4–phenyl–1,2,3,6–tetrahydropyridine; PCR Open array, PCR with Open Array technology.

The subchronic mouse model of PD was reproduced by repeatedly administering MPTP at gradually increasing doses from 8 to 40 mg/kg with a 24 h interval between injections [6]. The analysis of DA concentration and PCR with Open Array were done 24 h after the second injections of MPTP (10 mg/kg) and after the last one (40 mg/kg) (Figure 1).

### 2.1. The Concentration of Dopamine in the Striata of Mice after the Administration of 1-methyl-4-phenyl-1,2,3,6-tetrahydropyridine

**Figure 2:**
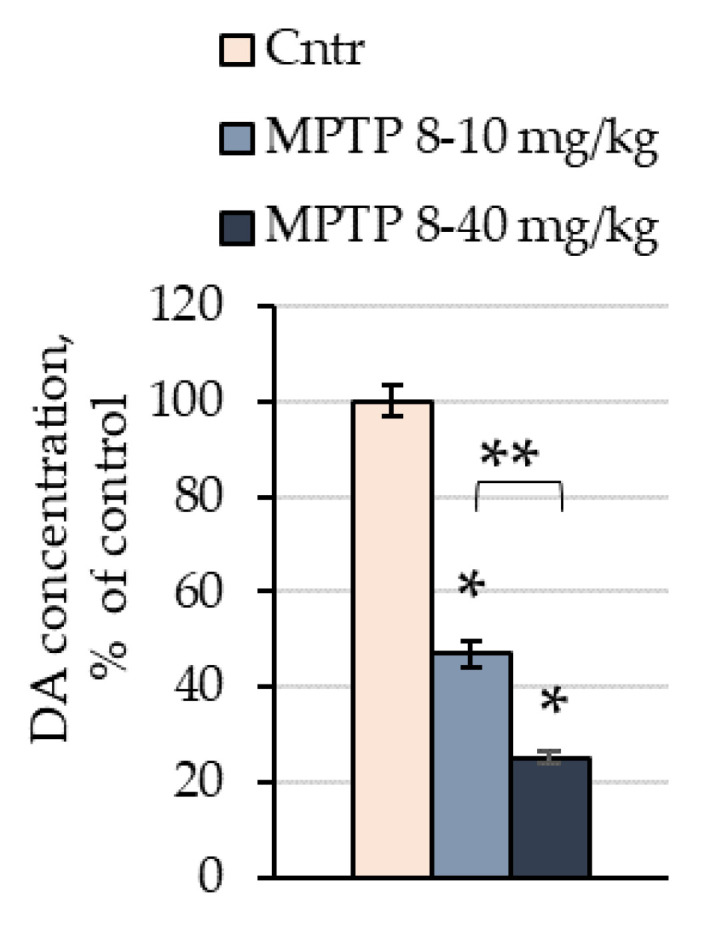
Dopamine concentration in the striatum of mice after two (8 and 10 mg/kg) and seven (8, 10, 12, 16, 20, 26, and 48 mg/kg) injections of 1-methyl-4-phenyl-1,2,3,6-tetrahydropyridine (MPTP) in relation to the control, which was taken as 100%. n = 8 per group. * *p* < 0.05, significant difference compared with the control (one-way ANOVA). ** *p* < 0.05, significant difference between the selected parameters (one-way ANOVA). Data are presented as mean ± SEM. Cntr, the control group that received 0.9% NaCl; DA, dopamine.

The concentration of DA in mice of the 1st group 24 h after two consecutive injections of MPTP at doses of 8 and 10 mg/kg, respectively, was reduced by 52% (46.9 pmol/mg) compared with the control (101.4 pmol/mg) (Figure 2). The concentration of DA in mice of the 2nd group 24 h after seven consecutive injections of MPTP at doses of 8, 10, 12, 16, 20, 26, and 40 mg/kg, in that order, decreased by 74% (25.2 pmol/mg) compared with the control (102.7 pmol/mg) (Figure 2).

### 2.2. Expression of Genes of Interest in the Substantia Nigra of Mice following Administration of 1-methyl-4-phenyl-1,2,3,6-tetrahydropyridine

All genes were divided into clusters by protein function: DA synthesis and degradation, DA reception, axonal transport and microtubules, synaptic vesicle cycle of neurotransmission, oxidative stress, protein degradation, inflammation and glial activation, and cell death. 

**Figure 3:**
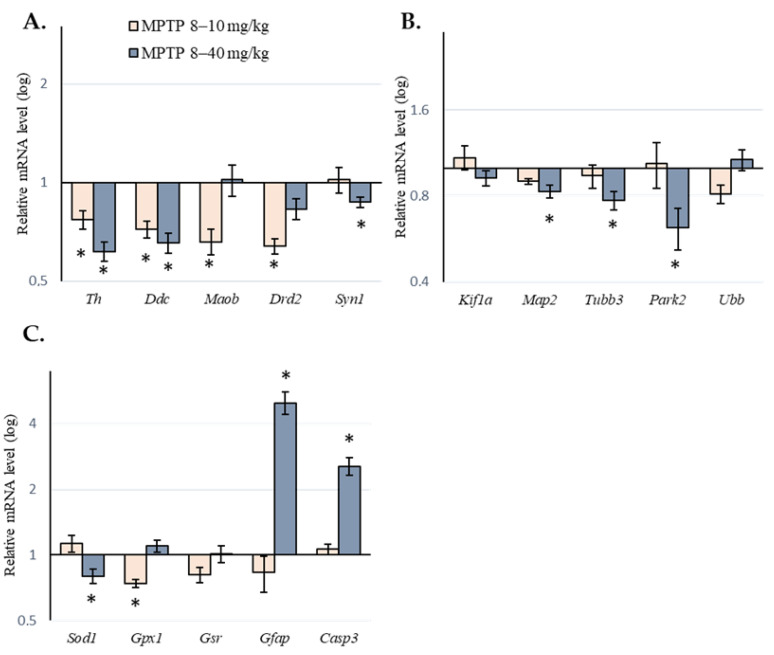
Relative expression of genes encoding the proteins involved in the synthesis, degradation, and reception of dopamine as well as in the synaptic vesicle cycle for neurotransmission (**A**); axonal transport and microtubules as well as protein degradation (**B**); oxidative stress, inflammation, and glial activation as well as cell death (**C**). Gene expression was assessed in the substantia nigra 24 h after the last of two injections of 1-methyl-4-phenyl-1,2,3,6-tetrahydropyridine (MPTP) at doses of 8 to 10 mg/kg and after the last of seven injections of MPTP at doses of 8 to 40 mg/kg. Data are shown as means with SEM. * *p* < 0.05, significant difference compared with the control (one-way ANOVA).

In mice of the 1st group, after two consecutive MPTP doses of 8 and 10 mg/kg, respectively, no increase in gene expression was found in any of the selected clusters. At the same time, there was a decrease in the gene expression of proteins associated with the following: synthesis and degradation of DA (*Th*, *Ddc*, and *Maob*); DA reception (*Drd2*); the synaptic vesicle cycle of neurotransmission (*Syt11*); oxidative stress (*Gpx1*). In other clusters, no changes in genes encoding proteins involved in axonal transport, protein degradation in the proteasome system, or cell death were found (Table 1 and Figure 3).

In mice of the 2nd group, after seven consecutive MPTP doses from 8 to 40 mg/kg, we found an increase in the gene expression of proteins associated with inflammation and activation of glia (*Gfap*) as well as cell death (*Casp3*, *Parp1*, *Aifm1*, *Cib1*, and *Ctsb*). In the same mice, we found a decrease in the gene expression of proteins associated with DA synthesis and degradation (*Th* and *Ddc*), axonal transport and microtubules (*Map2*, *Tubb3*, and *Tuba1a*), the synaptic vesicle cycle of neurotransmission (*Syn1*), protein degradation (*Park2* and *Psmc3*), oxidative stress (*Sod1* and *Keap1*), and inflammation and glial activation (*Akt1*) (Table 1 and Figure 3). The expression of some genes in each of the selected clusters did not change after the administration of MPTP (Table 1).

## 3. Discussion

At the first stage of this study, which aimed to assess the expression of genes encoding proteins specific for DAergic neurons as well as proteins involved in the molecular mechanisms of neurodegeneration in the SN as affected by PD, it was necessary to select a PD model that would reproduce the progressive degradation of the nigrostriatal system. Such a subchronic model of PD was recently developed in our laboratory in mice with systemic MPTP administration [6]. In this model, progressive degradation of the nigrostriatal DAergic system, which reproduced the continuous development of PD at the preclinical and clinical stages, was induced via repeated administration of MPTP at gradually increasing doses. MPTP was injected at intervals of 24 h, which corresponds to the time of neurodegeneration in response to a single injection of this neurotoxin. Two consecutive MPTP injections at doses of 8 mg/kg and 10 mg/kg reproduced the development of PD at the preclinical (asymptomatic) stage. Seven consecutive MPTP injections at doses of 8, 10, 12, 16, 20, 26, and 40 mg/kg, in that order, imitated the development of the clinical (symptomatic) stage of PD. In mice used in the subchronic PD model, as in patients with PD, the preclinical stage continuously passed to the clinical stage with the loss of 70–80% of DA in the striatum and the death of 38% nigral DAergic neurons. This was accompanied by the appearance of motor disorders [6]. In this study, we have accurately reproduced this model. The difference in the concentration of DA in the striatum in repeated experiments did not exceed 5%.

The value of the results obtained from a model of any disease depends on how well the model reproduces the pathological processes that develop in patients. Therefore, in the second stage of this study, we compared the expression of genes encoding proteins specific for DAergic neurons, as well as selected proteins involved in intercellular signaling and neurodegeneration in the SN, in our mouse model of subchronic PD (our results)to that found in patients in the clinical stage of PD (published literature data). In the absence of a preclinical PD diagnosis, postmortem material for such an analysis cannot be obtained from patients at the preclinical stage.

According to the research literature, analysis of gene expression in postmortem material was carried out both in the whole SN [7,8,9,10,11,12,13,14,15,16,17,18,19] and in individual DAergic neurons isolated by neuromelanin content [8,20,21,22,23,24]. Our data on gene expression in the SN in mice in the PD model were compared with the published data obtained from the SN of patients but not with those obtained from individual neuromelanin-containing neurons (Table 2) because pathological material from patients can be obtained only many years after diagnosis and treatment. Obviously, the treatment itself can change the gene expression of proteins involved in pathological processes. Indeed, Tiklova and co-authors [25] have shown that gene expression is significantly different between the early and late clinical stages of PD. In addition, researchers often do not provide information on patient treatments that can affect gene expression in the nigrostriatal system [26,27]. However, despite the difficulties in interpreting changes in gene expression in PD patients, this is the only approach that can be used to validate animal models, although with great caution. 

Of all of the genes we analyzed in the SN of mice in the clinical stage model, only 20 genes were also analyzed in the SN of patients with PD (Table 2). For 7 out of 20 genes in mice and patients, we found either a unidirectional change in expression or no change (*Th*, *Ddc*, *Map2*, *Snca*, *Psmc3*, *Casp3*, and *Mapk8*). For 13 genes, opposite changes in gene expression were observed (*Maoa*, *Dynll1*, *Mapt*, *Syt11*, *Syt1*, *Nsf*, *Park2*, *Ube2n*, *Psmd4*, *Ubb*, *Gfap*, *Vps35*, and *Ctsb*). As for the genes with unidirectional expression in the SN in the mouse model of the clinical stage of PD and in patients with PD, they are present in almost all of the selected gene clusters except for “Oxidative stress” and “Inflammation and glial activation” (Table 2).

Changes in the expression of genes for proteins of the DAergic phenotype (*Th* and *Ddc* of the “DA synthesis and degradation” cluster) in the SN in our model of the clinical stage of PD coincided with changes in the expression of the same genes in patients with PD. In both cases, the expression of these genes was reduced [7,14,28]. It should be noted that in mice in the acute model of the clinical stage of PD and in patients with PD, a direct relationship was shown between the expression of the tyrosine hydroxylase gene and the level of the tyrosine hydroxylase protein in the SN [29,30]. The content of tyrosine hydroxylase, as well as its activity in surviving neurons, remained at the control level [29,30,31]. It is possible that the decrease in the gene expression of proteins characteristic of DAergic neurons that we found in this study is associated with a loss of DAergic neurons.

It is believed that the death of neurons in PD begins with a degradation of axonal terminals and followed by a retrograde spread of degradation up to the cell bodies, which is accompanied by impaired axonal transport [32]. However, it is still not clear what the primary cause of neuronal death in PD is, whether it is the impaired functioning of axonal terminals or impaired axonal transport [33,34,35]. Considering that analysis of gene expression in PD patients is carried out on pathological material obtained many years after the diagnosis of PD (by the appearance of motor symptoms) and the start of pharmacotherapy, it is impossible to determine which gene expressions change at an early clinical stage before the start of patient treatment. However, in the SN both in our mouse model of the clinical stage of PD (see Results) and in postmortem PD patients, a decrease in the expression of *Map2*, a microtubule stabilizing protein, was found [16]. These data suggest that the assembly/disassembly of microtubules is impaired at the initial stage of neurodegeneration, which leads to a disorganization of axonal transport and of the functioning of synaptic terminals. The decreased expression of genes for microtubule stabilizing proteins such as β-tubulin, α-tubulin, synapsin-1, and parkin [36] found in the SN in our mouse model of the clinical stage of PD supports this assumption. Despite the fact that analysis of the gene expression of microtubule stabilizing proteins has not been performed in PD patients, our experimental data are a strong argument in favor of the assumption that neurodegeneration begins with a degradation of microtubules.

An important characteristic of the pathogenesis of PD is the formation of Lewy bodies, which contain proteins such as aggregated α-synuclein, tau protein, and ubiquitin [37,38]. Despite the accumulation of α-synuclein in patients with PD, most studies have shown that the expression of the α-synuclein gene does not change or even decrease compared with age-related controls [14,16,17]. This result is confirmed in our mouse model of the clinical stage of PD. Indeed, we did not find any changes in the expression of the *Snca* gene in these animals. It was shown that the aggregation of α-synuclein and its transformation first into oligomeric neurotoxic complexes and then into Lewy bodies is the result of impaired degradation of α-synuclein by the ubiquitin–proteasome system [39,40,41]. This is confirmed by our data, which details a decrease in the expression of the *Psmc3* gene that encodes one of the proteasome subunits in the SN in our model of the clinical stage of PD. This has also been shown in patients with PD [15]. It is important to note that one of the targets for the toxic action of oligomeric α-synuclein is constituted by microtubules, and that their degradation disrupts axonal transport. Although oxidative stress is believed to be the trigger for α-synucleinopathy [42], we have not found any unidirectional changes in the expression of genes for antioxidant system enzymes that regulate the expression of these genes in the SN in mice in our model of the clinical stage of PD (see Results) and in patients with PD.

Despite a great interest in the death mechanisms of DAergic neurons of the nigrostriatal system in PD (apoptosis, necrosis, or autophagy), this question still remains open. Research literature data on this issue are contradictory. However, some studies claim that DAergic neurons die mainly due to apoptosis [43,44,45,46,47,48,49]. Evidence that apoptosis is the main death mechanism of DAergic neurons is constituted by an increased expression of active caspase 3, the main inducer of apoptosis [45,46]. An increase in *Casp3* gene expression was confirmed in our subchronic model of the clinical stage of PD, which is consistent with the data obtained in other subchronic and chronic models that show neuronal death through apoptosis [50,51,52]. In addition, both in patients with PD [16] and in our model of the clinical stage of PD, no changes in the expression of the *Mapk8* gene, a protein that triggers autophagy, were found in the SN.

There are only a few studies evaluating gene expression in the SN when modeling PD in non-human primates using MPTP [53]. Of the seven genes evaluated by Ohnuki et al. (2010) and in our study (*Th*, *Ddc*, *Ubb*, *Gfap*, *Drd2*, *Mapt*, and *Tubb3*), the same changes in the expression of only four genes (*Th*, *Ddc*, *Ubb*, and *Gfap*) were observed. In addition, it was shown that changes in the expression of the *Mapt* gene in the SN of non-human primates in the PD model and in the SN postmortem in PD patients coincide, whereas the changes in the expression of *Ubb* and *Gfap* genes differ. It follows that both of the above models do not fully reproduce the neurodegenerative changes in the SN that are characteristic of PD patients. Nevertheless, both models of PD are useful and complement each other in studying the mechanisms of the neurodegeneration and neuroplasticity of the nigrostriatal dopaminergic system.

Considering that the SN contains both neurons and glial cells, we have evaluated in the mouse model of the clinical stage of PD genes that are predominantly expressed in neurons. This has made it possible to compare our data with those obtained on neuromelanin-containing cells isolated via microdissection from the SN of PD patients (Table 3).

It was shown that the expression of the five genes characteristic of neurons did not change in the SN in our mouse model of the clinical stage of PD (Table 3). These data are partly consistent with the study of Zaccaria [22], who did not find changes in the expression of three neuronal genes (*Drd2*, *Kif1a*, and *Kif5a*) in the SN of patients, but they do not agree with the data obtained in other similar studies [20,21,24]. As noted earlier, discrepancies in the published literature data on gene expression in patients with PD may be due to the use of pathological material obtained from patients with varying degrees of pathology as well as with different histories of pharmacotherapy [25,54]. Our failure to detect changes in the expression of kinesin genes (kinesins 1a and 5a), axonal transport proteins, and synaptotagmin 1, a vesicular cycle protein, in the SN of mice in our model of the clinical stage of PD supports our assumption that neurodegeneration is associated with impaired axonal transport and neurotransmission. It is possible that a disruption of the vesicular cycle in neurodegeneration predominates over a disruption of axonal transport. This is evidenced by the fact that all available studies show a decrease in the expression of the synaptotagmin 1 and 11 genes [20,21,22,23,24], whereas this is not always the case for kinesin gene expression [20,22,24].

Given that there is no data on gene expression in the nigrostriatal system at the preclinical stage in patients with PD due to a lack of preclinical diagnoses, the only way to conduct such studies is through the use of experimental models.

The development of a subchronic model of PD, which reproduces the progressive degradation of the nigrostriatal system, has made it possible to evaluate the expression of genes encoding DA-synthesizing enzymes and proteins involved in the molecular mechanisms of neurodegeneration in the SN in mice in models of the preclinical and clinical stages of PD (Table 4).

According to the expression of genes in the SN of mice in our models of the preclinical and clinical stages, these genes can be divided into two groups. The first group includes genes whose expression does not change in the model of the preclinical stage but changes in the model of the clinical stage of PD compared with the control. The second group includes genes whose expression is downregulated in the model of the preclinical stage but does not change in the model of the clinical stage of PD compared with the control. 

In the first case, changes in gene expression indicate the progression of the neurodegenerative process during the transition from the preclinical to the clinical stage. This is characteristic of genes encoding proteins involved in axonal transport (*Map2*, *Tubb3*, and *Tuba1a*), synaptic vesicle cycle and neurotransmission (*Syn1*), inflammation and glial activation (*Gfap*), and cell death mechanisms (*Casp3,* and *Parp1*). It should be noted that the decrease in the gene expression of some neuronal proteins in the SN (e.g., *Th*) in mice in the clinical stage model could be due to the death of DAergic neurons rather than downregulation of gene expression [54]. In the second case, a change in gene expression could indicate early neurodegenerative processes that develop in the SN at the preclinical stage. An example is the decrease in the expression of the *Drd2* (DA reception), *Syt11* (synaptic vesicle cycle of neurotransmission), and *Gpx1* (antioxidant system) genes in the model of the preclinical stage of PD. Considering that D2 receptors are autoreceptors [55] and that synaptotagmin 11 (*Syt11*) is a non-Ca2+-dependent protein that regulates endocytosis [56], the detected changes may suggest a reorganization of the chemical machinery of DA release and uptake.

## 4. Materials and Methods

### 4.1. Animals 

Male C57BL/6 mice aged 8–12 weeks and weighing 22–25 g (n = 32) were used in this study. The animals were housed at 21–23 °C in a 12h light–dark cycle with free access to food and water. Experimental procedures were carried out in accordance with the National Institutes of Health Guide for the Care and Use of Laboratory Animals (8th edition, 2011) and were approved by the Animal Care and Use Committee of the Koltzov Institute of Developmental Biology of the Russian Academy of Sciences (protocol №50 from 5 August 2021).

Two groups of mice were used, which were subcutaneously injected with either MPTP (Sigma-Aldrich, USA) or 0.9% NaCl (control). In the first group, mice (n = 8) were sequentially injected with MPTP at increasing doses of 8 and 10 mg/kg at 24-h intervals, and in the second group (n = 8), mice were sequentially injected with MPTP at increasing doses of 8, 10, 12, 16, 20, 26, and 40 mg/kg at 24-h intervals. Mice in the control groups were injected with 0.9% NaCl according to the same schemes (n = 8 for each control group). Materials for analysis were obtained 24 h after the last injection (Figure 1). 

### 4.2. Sample Preparation for Analysis

Mice from all groups in the experiment and in the control were decapitated under isoflurane anesthesia (Baxter, Deerfield, IL, USA), and the brains were removed and cut along the middle sagittal plane. The striatum was excised from one cerebral hemisphere from bregma 1.70 to bregma 0.14 in the rostrocaudal direction according to the atlas [57], and the SN was excised from bregma −2.54 to bregma −4.04 using a dissecting microscope (Leica M60, Wetzlar, Germany). This procedure was described in detail earlier [31,58]. The obtained tissue samples from mice were weighed, frozen in liquid nitrogen, and stored at −70 °C until further analysis.

### 4.3. Methods

#### 4.3.1. High Performance Liquid Chromatography with Electrochemical Detection

HPLC-ED was used to determine the concentration of DA in the striatum samples of mice from all groups. The tissue samples were homogenized using an ultrasonic homogenizer (UP100H, Hielscher Ultrasonics GmbH, Teltow, Germany) in 0.1 N HClO_4_ (Sigma-Aldrich, St. Louis, MO, USA) in a solution containing the internal standard 3,4-dihydroxybenzylamine hydrobromide (Sigma-Aldrich, USA) at a concentration of 250 pmol/mL. This mixture was centrifuged at 2000× *g* for 20 min.

The separation of DA was carried out on a ReproSil-Pur reversed-phase column, ODS-3, 4 × 100 mm with a pore diameter of 3 µm (Dr. Majsch, Ammerbuch, Germany) at a temperature of +30 °C and a mobile phase speed of 1 mL/min and supported by an LC-20ADsp liquid chromatograph (Shimadzu, Kyoto, Japan). The mobile phase included: 0.1 M citrate–phosphate buffer, 0.3 mM sodium octanesulfonate, 0.1 mM EDTA, and 9% acetonitrile (all reagents from Sigma-Aldrich, St. Louis, MO, USA), pH 2.5. A Decade II electrochemical detector (Antec Leyden, Leuden, The Netherlands) was equipped with a working glassy carbon electrode (+0.85 V) and an Ag/AgCl reference electrode. Peaks of DA and the internal standard were identified by their release time in the standard solution. The content of analytes was calculated by using the internal standard method as the ratio of the peak areas of DA standards to the peak areas of these substances in a biological sample using the LabSolutions software (Shimadzu, Japan). Striatum samples were normalized to tissue weight. 

#### 4.3.2. Real-Time PCR 

The extraction of total RNA from the SN samples of mice from all groups was carried out in 1 mL TRI-reagent (Sigma-Aldrich, USA) by pipetting according to the manufacturer’s instructions. For better RNA precipitation, 1 µg glycogen (Thermo Fisher Scientific, Waltham, MA, USA) was added to each sample. The concentration of total RNA in the samples was determined using Nanodrop 8000 (Thermo Scientific, USA). Total RNA was treated with RNase-free DNase I (Thermo Fisher Scientific, USA) to remove residual genomic DNA. 0.5 µg RNA was taken into reverse transcription for cDNA synthesis from the SN samples. Reverse transcription was performed using the RevertAid H Minus First Strand cDNA Synthesis Kit (Thermo Fisher Scientific, USA) with random hexamer primers in accordance with the manufacturer’s instructions (Thermo Fisher Scientific, USA). The reaction continued for 60 min at 42 °C and was stopped by heating for 10 min at 70 °C, which was followed by cooling the samples on ice. 

Real-time PCR analysis was performed using Open Array technology with TaqMan Open Array RT PCR Custom Format 112 chips (Lot 37B6745, REF 4,470,756 Applied Biosystems, Waltham, MA, USA) (Table 5 and Table S1), which allowed the determination of the expression of 81 genes of interest in each of our samples. For PCR, 250 ng/µL cDNA was taken for segments with 56 cells. The results were processed using the QuantStudio 12K Flex software (Applied Biosystems, USA) and Excel (Microsoft Office, Redmond, WA, USA). Gene expression levels are expressed as 2^–ΔΔCt^ values normalized to the level of *Cyc1* as a house-keeping gene. Formulas (1) and (2) were used for calculating ΔΔCt as follows:

∆∆Ct = (∆Ct(sample) − ∆Ct(medium control)), where(1)

∆Ct = (Ct(gene) − Ct(Cyc1).(2)

The results were calculated as the geometric mean of the group [59] and are presented as fold changes with respect to the control, where the ∆∆Ct of the control group is taken as 1.

Lists of genes and their target names as well as genes not expressed in the SN are presented in Tables S1 and S2 in the Supplementary Materials.

The gene names were taken from the National Library of Medicine’s GenBank database (https://www.ncbi.nlm.nih.gov/genbank, accessed on 1 December 2022).

#### 4.3.3. Statistical Analysis

Data are presented as the means with SEM. For the analysis, we used the one-way ANOVA test followed by Tukey’s post hoc test for multiple comparisons, employing the GraphPad Prism 6.0 software package (GraphPad Software, La Jolla, CA, USA). *p* ˂ 0.05 was considered a significant difference.

## 5. Conclusions

The fight against neurodegenerative diseases such as Alzheimer’s disease and PD is one of the global challenges of the 21st century. All attempts to cure these patients have so far been unsuccessful. This is due to the fact that neurodegenerative diseases develop for decades at the preclinical stage without the manifestation of diagnostic clinical symptoms and, hence, without treatment. Therefore, one of the highest priorities in neuroscience is the development of preclinical diagnosis techniques and preventive neuroprotective therapy, which would slow down the death of neurons and, thus, prolong the period of a patient’s comfortable (asymptomatic) life. Success in the development of these technologies depends on our knowledge of the molecular mechanisms of pathogenesis, primarily at the preclinical stage. In the absence of preclinical diagnosis, the mechanisms of pathogenesis can only be studied on animal models but not in patients. Therefore, our study was devoted to the evaluation of some molecular mechanisms of PD pathogenesis on a subchronic model that we developed in mice using MPTP, a neurotoxin of the DAergic neurons that control motor function. This model reproduces the progressive development of PD with a gradual transition from the preclinical stage to the clinical one. In this study, we evaluated the gene expression of proteins involved in the functioning of DAergic neurons and neurodegeneration in the mouse SN, the site of localization of these neurons. We found that in the SN in our mouse model of the preclinical stage of PD, there were changes in the expression of a number of genes encoding enzymes of the synthesis and degradation of DA as well as proteins involved in the vesicular cycle and neurotransmission, axonal transport, degradation of proteins in the ubiquitin–proteasome system, neuroinflammation, and cell death. Similar changes in gene expression were demonstrated in previous studies in patients at the clinical stage of PD (postmortem), indicating good reproducibility of PD pathogenesis in our model. Further comparative analysis of gene expression in the SN in our mouse PD models showed that the expression of some genes changed in the model of the preclinical stage of PD, thereby preceding the death of dopaminergic neurons. Thus, this study of the gene expression of proteins involved in the functioning of DAergic neurons and neurodegeneration in the SN in our MPTP mouse model of PD opens up new opportunities for an in-depth study of the molecular mechanisms of the pathogenesis of this disease and the development of preventive neuroprotective therapies.

## Figures and Tables

**Table 1:** Changes in the gene expression of proteins of the dopaminergic phenotype, as well as proteins involved in synaptic neurotransmission and neurodegeneration, 24 h after the last administration of 1-methyl-4-phenyl-1,2,3,6-tetrahydropyridine (MPTP) at increasing doses from 8 to 10 mg/kg and from 8 to 40 mg/kg. The data are presented as ratios of the level of gene expression in the experimental group to the level of gene expression in the control, which was taken as 1.

Gene	Protein	Function	Scheme of Subcutaneous Administration of MPTP, mg/kg			
			8–10		8–40	
			Fold Change	*p*	Fold Change	*p*
**Dopamine synthesis and degradation**						
*Th*	Tyrosine hydroxylase	DA synthesis	0.77 *	0.01	0.64 *	0.01
*Ddc*	Aromatic L-amino acid decarboxylase	DA synthesis	0.72 *	0.01	0.69 *	0.01
*Maoa*	Monoamine oxidase A	DA degradation	**1.18**	0.37	**1.06**	0.99
*Maob*	Monoamine oxidase B	DA degradation	0.66 *	0.01	**1.02**	0.98
*Comt*	Catechol-O-methyltransferase	DA degradation	**0.87**	0.99	**1.05**	0.99
**Dopamine reception**						
*Drd2*	Dopamine receptor 2	DA reception	0.64 *	0.01	**0.83**	0.37
**Axonal transport and microtubules**						
*Kif1a*	Kinesin family member 1A	Axonal transport	**1.09**	0.95	**1.00**	0.99
*Kif1b*	Kinesin family member 1B	Axonal transport	**0.98**	0.99	**1.17**	0.99
*Kif5a*	Kinesin family member 5A	Axonal transport	**0.89**	0.88	**1.14**	0.62
*Dync1h1*	Dynein cytoplasmic 1 heavy chain 1	Axonal transport	**1.17**	0.69	**0.90**	0.97
*Dynll1*	Dynein light chain	Axonal transport	**0.85**	0.72	**1.07**	0.98
*Dctn1*	Dynactin 1	Axonal transport	**1.09**	0.87	**1.04**	0.99
*Map2*	Microtubule-associated protein 2	Axonal transport	**0.90**	0.61	0.83 *	0.04
*Mapt*	Microtubule-associated protein tau	Axonal transport	**1.15**	0.36	**0.95**	0.98
*Mark2*	MAP/microtubule affinity regulating kinase 2	Axonal transport	**0.97**	0.99	**1.49**	0.13
*Tubb3*	β-tubulin	Axonal transport	**0.94**	0.94	0.77 *	0.02
*Tuba1a*	α-tubulin	Axonal transport	**0.98**	0.90	0.74 *	0.04
**Synaptic vesicle cycle of neurotransmission**						
*Snca*	α-Synuclein	Neurotransmission	**0.78**	0.18	**0.93**	0.90
*Syn1*	Synapsin 1, SNARE-complex	Vesicular cycle	**1.02**	0.98	0.70 *	0.01
*Stx1a*	Syntaxin 1A	Vesicular cycle	**0.76**	0.96	**1.17**	0.94
*Syt1*	Synaptotagmin I	Vesicular cycle	**0.89**	0.75	**0.79**	0.12
*Syt11*	Synaptotagmin 11	Endocytosis	0.72 *	0.02	**0.87**	0.05
*Rab5a*	RAB5A	Endocytosis	**0.89**	0.49	**0.92**	0.60
*Rab7*	RAB7	Endocytosis	**0.93**	0.98	**1.03**	0.98
*Nsf*	N-Ethylmaleimide sensitive fusion protein	Vesicular cycle	**0.92**	0.96	**0.96**	0.98
*Dnm1*	Dynamin 1-like protein	Vesicular cycle	**0.81**	0.48	**1.40**	0.13
**Protein degradation**						
*Park2*	Parkin	Ubiquitinylation	**1.04**	0.90	0.62 *	0.01
*Uba3*	Ubiquitin-like modifier activating enzyme 3	Ubiquitinylation	**0.82**	0.49	**1.21**	0.72
*Usp47*	Ubiquitin specific peptidase 47	Ubiquitinylation	**0.79**	0.79	**1.62**	0.98
*Ubb*	Ubiquitin B	Ubiquitinylation	**0.81**	0.16	**1.07**	0.71
*Ube2n*	Ubiquitin-conjugating enzyme E2N	Ubiquitinylation	**0.83**	0.49	**0.92**	0.72
*Psmb4*	Proteasome 20S Subunit Beta 4	Protein degradation	**0.93**	0.95	**0.88**	0.45
*Psmc3*	Proteasome 26S subunit ATPase 3	Protein degradation	**1.07**	0.89	0.76 *	0.02
*Psmd4*	Proteasome 26S subunit non-ATPase 4	Protein degradation	**0.87**	0.99	**1.04**	0.83
**Oxidative stress**						
*Sod1*	Superoxide dismutase 1	Antioxidant system	**1.13**	0.60	0.80 *	0.05
*Gpx1*	Glutathione peroxidase 1	Antioxidant system	0.74 *	0.04	**1.10**	0.72
*Gsr*	Glutathione reductase	Antioxidant system	**0.81**	0.07	**1.01**	0.87
*Txnrd1*	Thioredoxin reductase 1	Antioxidant system	**0.80**	052	**1.09**	0.99
*Nos1*	Nitric oxide synthase 1, neuronal	Antioxidant system	**0.61**	0.72	**1.02**	0.99
*Prdx1*	Peroxiredoxin 1	Antioxidant system	**0.81**	0.65	**0.93**	0.74
*Nfe2l2*	Nuclear factor erythroid 2-related factor 2	Regulation of antioxidant system	**1.14**	0.92	**1.03**	1.00
*Keap1*	Kelch-like ECH-associated protein 1	Regulation of antioxidant system	**1.31**	0.45	0.60 *	0.04
*Sigmar1*	Sigma-1 receptor	Chaperone protein	**0.97**	0.99	**1.09**	0.88
**Inflammation and glial activation**						
*Gfap*	Glial fibrillary acidic protein	Glial activation	**0.83**	0.99	4.99 *	0.01
*Akt1*	Protein kinase B alpha	Inflammatory signaling pathway	**0.83**	0.48	0.81 *	0.02
*Tgfb1*	Transforming growth factor, beta 1	Anti-inflammatory cytokine	**0.75**	0.65	**1.35**	0.65
**Cell death**						
*Casp3*	Caspase 3	Apoptosis	**1.06**	0.99	2.55 *	0.01
*Parp1*	Poly [ADP-ribose] polymerase 1	Apoptosis	**1.01**	0.96	1.72 *	0.02
*Aifm1*	Apoptosis inducing factor mitochondria associated 1	Apoptosis	**1.08**	0.99	1.62 *	0.04
*Bcl2l11*	BCL2 like 11	Apoptosis	**0.82**	0.8	**1.38**	0.33
*Map3k5*	Mitogen-activated protein kinase kinase kinase 5	Apoptotic signaling	**0.64**	0.89	**0.95**	0.65
*Cib1*	Calcium and integrin binding 1	Apoptotic signaling	**0.94**	0.96	2.55 *	0.01
*Bax*	Bax protein	Apoptosis	**0.90**	0.94	**1.17**	0.60
*Trp53*	Transformation related protein 53	Apoptosis	**1.04**	0.99	**0.10**	0.99
*Fos*	FBJ osteosarcoma oncogene	Apoptosis	**0.91**	0.99	**1.44**	0.07
*Vps35*	Vacuolar protein sorting-associated protein 35	Autophagy	**0.87**	0.88	**1.05**	0.99
*Mapk8*	Mitogen-activated protein kinase 8	Autophagy	**1.03**	0.95	**0.85**	0.95
*Lamp2*	Lysosomal-associated membrane protein 2	Autophagy	**0.83**	0.59	**0.87**	0.70
*Atg16l1*	Autophagy related 16-like 1 (S. cerevisiae)	Autophagy	**0.87**	0.91	**0.87**	0.77
*Atg5*	Autophagy related 5	Autophagy	**0.77**	0.65	**1.00**	0.99
*Ctsb*	Cathepsin B	Necrosis	**0.85**	0.52	1.31 *	0.01
*Capn1*	Calpain 1	Necrosis	**1.16**	0.78	**1.19**	0.98
*Eif2ak3*	Endoplasmic reticulum (ER) to nucleus signaling 2	ER stress	**0.86**	0.92	**1.53**	0.31
*Atf6*	Activating transcription factor 6	ER stress	**1.18**	0.78	**1.02**	0.99

Gene names were taken from the National Library of Medicine GenBank (https://www.ncbi.nlm.nih.gov/genbank, accessed on 1 December 2022). Significant differences in gene expression relative to the control (taken as 1) are highlighted in color and in bold: red: decrease (less than 1), green: increase (greater than 1). Data are shown as the mean. * *p* < 0.05, significant difference compared with the control (one-way ANOVA); n = 8. DA, dopamine; ER, endoplasmic reticulum; MPTP, 1-methyl-4-phenyl-1,2,3,6-tetrahydropyridine.

**Table 2:** Comparison of gene expression for the dopaminergic phenotype, synaptic neurotransmission, and neurodegeneration proteins in the substantia nigra of mice in the clinical stage of Parkinson’s disease model (CSSM) and in the substantia nigra of patients with Parkinson’s disease (SN PD).

Gene	Protein	Function	CSSM	SN PD	Articles
**Dopamine synthesis and degradation**					
*Th*	Tyrosine hydroxylase	DA synthesis	↓	↓	[7]
*Ddc*	Aromatic L-amino acid decarboxylase	DA synthesis	↓	↓	[7,14]
*Maoa*	Monoamine oxidase A	DA degradation	→	↑	[15]
**Axonal transport and microtubules**					
*Dynll1*	Dynein light chain	Axonal transport	→	↓	[14,17]
*Mapt*	Tau protein	Axonal transport	→	↓	[15,19]
*Map2*	Microtubule-associated protein 2	Axonal transport	↓	↓/→	[14,16]
**Synaptic vesicle cycle of neurotransmission**					
*Snca*	α-Synuclein	Neurotransmission	→	↓/→	[14,16,17]
*Syt11*	Synaptotagmin 11	Endocytosis	→	↓	[11]
*Syt1*	Synaptotagmin 1	Vesicular cycle	→	↓	[10,12,13,17]
*Nsf*	N-Ethylmaleimide sensitive fusion protein	Vesicular cycle	→	↓	[12,13,14,17]
**Protein degradation**					
*Park2*	Parkin	Ubiquitinylation	↓	→	[16]
*Ube2n*	Ubiquitin-conjugating enzyme E2N	Ubiquitinylation	→	↓	[15]
*Ubb*	Ubiquitin B	Ubiquitinylation	→	↓	[11,15]
*Psmc3*	Proteasome 26S subunit ATPase 3	Protein degradation	↓	↓	[15]
*Psmd4*	Proteasome 26S subunit non-ATPase 4	Protein degradation	→	↑	[15]
**Inflammation and glial activation**					
*Gfap*	Glial fibrillary acidic protein	Glial activation	↑	↓	[11]
**Cell death**					
*Casp3*	Caspase 3	Apoptosis	↑	↑	[16]
*Vps35*	VPS35 retromer complex component	Autophagy	→	↓	[11]
*Mapk8*	Mitogen-activated protein kinase 8	Autophagy	→	→	[16]
*Ctsb*	Cathepsin B	Necrosis	↑	↓	[11]

↑—increase, ↓—decrease, →—no deference in gene expression compared to the control. Blue: the same changes in gene expression in models of both stages of Parkinson’s disease.

**Table 3:** Comparison of changes in neuronal protein gene expression in the substantia nigra of mice in our clinical stage model of Parkinson’s disease (CSSM) and in neuromelanin-containing neurons isolated from the substantia nigra of patients with Parkinson’s disease (NMPC).

Gene	Protein	Function	CSSM	NMPC	Articles
**Dopamine reception**					
*Drd2*	Dopamine receptor 2	DA reception	→	↑/→	[21,22,23]
**Axonal transport and microtubules**					
*Kif1a*	Kinesin family member 1A	Axonal transport	→	↓/→	[20,22]
*Kif5a*	Kinesin family member 5A	Axonal transport	→	↓/→	[22,24]
*Mark2*	MAP/microtubule affinity regulating kinase 2	Axonal transport	→	↑/↓	[23,24]
**Synaptic vesicle cycle of neurotransmission**					
*Syt1*	Synaptotagmin 1	Vesicular cycle	→	↓	[20,21,22,23,24]

↑—increase, ↓—decrease, →—no deference in gene expression compared to the control. Blue, the same changes in gene expression in models of both stages of Parkinson’s disease.

**Table 4:** Gene expression of enzymes of dopamine metabolism and reception as well as proteins involved in neurodegeneration in the substantia nigra in mice in preclinical (PSSM) and clinical (CSSM) models of Parkinson’s disease.

Gene	Protein	Protein Function	Subchronic Model Stages:	
			PSSM	CSSM
**Dopamine synthesis and degradation, reception**				
*Th*	Tyrosine hydroxylase	DA synthesis	↓	↓
*Ddc*	Aromatic L-amino acid decarboxylase	DA synthesis	↓	↓
*Maob*	Monoamine oxidase B	DA degradation	↓	→
**Dopamine reception**				
*Drd2*	Dopamine receptor 2	DA reception	↓	→
**Axonal transport and microtubules**				
*Map2*	Microtubule-associated protein 2	Axonal transport	→	↓
*Tubb3*	β-tubulin	Axonal transport	→	↓
***Tuba1a***	α-tubulin	Axonal transport	→	↓
**Synaptic vesicle cycle of neurotransmission**				
***Syn1***	Synapsin 1	Vesicular cycle	→	↓
*Syt11*	Synaptotagmin 11	Endocytosis	↓	→
**Protein degradation**				
*Park2*	Parkin	Ubiquitinylation	→	↓
*Psmc3*	Proteasome 26S subunit ATPase 3	Protein degradation	→	↓
**Oxidative stress**				
***Gpx1***	Glutathione peroxidase 1	Antioxidant system	↓	→
***Keap1***	Kelch-like ECH-associated protein 1	Regulation of antioxidant system	→	↓
**Inflammation and glial activation**				
***Gfap***	Glial fibrillary acidic protein	Glial activation	→	↑
*Akt1*	Protein kinase B alpha	Inflammatory signaling pathway	→	↓
**Cell death**				
***Casp3***	Caspase 3	Apoptosis	→	↑
***Parp1***	Poly [ADP-ribose] polymerase 1	Apoptosis	→	↑
*Aifm1*	Apoptosis inducing factor mitochondria associated 1	Apoptosis	→	↑
***Cib1***	Calcium and integrin binding 1	Apoptotic signaling	→	↑
***Ctsb***	Cathepsin B	Necrosis	→	↑

↑—increase, ↓—decrease, →—no deference in gene expression compared to the control. Green, change in gene expression in models of both stages of Parkinson’s disease; Pink, change in gene expression only in the model of the preclinical stage of Parkinson’s disease; Grey, change in gene expression only in the model of the clinical stage of Parkinson’s disease.

**Table 5:** List of genes presented on chips produced by Thermo Fisher Scientific for RT PCR with Open Array Technology and clustered by protein function.

Cluster of Genes	Genes
Dopamine synthesis and degradation	*Th*, *Ddc*, *Dbh*, *Pnmt*, *Maoa*, *Maob*, and *Comt*
Dopamine reception	*Drd1–Drd5*
Axonal transport and microtubules	*Kif1a*, *Kif1b*, *Kif5a*, *Kif2c*, *Dync1h1*, *Dynll1*, *Dctn1*, *Mapt*, *Map2*, *Mark2*, *Tubb3*, and *Tuba1a*
Synaptic vesicle cycle of neurotransmission	*Snca*, *Syn1*, *Stx1a*, *Syt1*, *Syt11*, *Rab5a*, *Rab7*, *Nsf*, *Dnm1l*, and *Vps35*
Oxidative stress	*Sod1*, *Gpx1*, *Gsr*, *Txnrd1*, *Nos1*, *Prdx1*, *Nfe2l2*, *Agtr2*, *Keap1*, and *Sigmar1*
Protein degradation	*Park2*, *Ube2n*, *Uba3*, *Psmb4*, *Psmc3*, *Psmd4*, *Usp47*, *Ubb*, *Cacna1d*, and *Trpm2*
Inflammation and glial activation	*Calb1*, *Ifng*, *Tgfb1*, *Akt1*, *Cnr1*, *Ptgs2*, *Traf1*, and *Cxcl11*
Cell death	*Casp1*, *Casp3*, *Parp1*, *Aifm1*, *Bcl2l11*, *Map3k5*, *Cib1*, *Trp53*, *Bax*, *Fos*, *Mapk8*, *Lamp2*, *Atg16l1*, *Atg5*, *Tnf*, *Ctsb*, *Ern2*, *Eif2ak3*, and *Atf6*

## Data Availability

The data presented in this study are available on request from the corresponding author. The data are not publicly available due to legal issues.

## Supplementary Materials

The following supporting information can be downloaded at: https://www.mdpi.com/article/10.3390/ijms24033027/s1.

## Abbreviations

DA	dopamine
DAergic	dopaminergic
MPTP	1–methyl–4–phenyl–1,2,3,6–tetrahydropyridine
PD	Parkinson’s disease
SN	substantia nigra
