# Supplementary material for: The Gene Expression of Proteins Involved in Intercellular Signaling and Neurodegeneration in the Substantia Nigra in a Mouse Subchronic Model of Parkinson’s Disease
Source: Int J Mol Sci. 2023 Feb 3;24(3):3027. doi: 10.3390/ijms24033027 (PMC9917821; doi:10.3390/ijms24033027)
Supplement: Supplementary file 1 [file ijms-24-03027-s001.zip › ijms-2112036-supplementary.pdf]

Table S1. Genes and their target names on PCR chips for Open Array technology

No	Gene	Target Name	Reporter
**1.**	*Gfap*	Mm01253033_m1	FAM
**2.**	*Cyc1*	Mm00470540_m1	FAM
**3.**	*Th*	Mm00447557_m1	FAM
**4.**	*Ddc*	Mm00516688_m1	FAM
**5.**	*Dbh*	Mm00460472_m1	FAM
**6.**	*Pnmt*	Mm00476993_m1	FAM
**7.**	*Maoa*	Mm00558004_m1	FAM
**8.**	*Maob*	Mm00555412_m1	FAM
**9.**	*Comt*	Mm00514377_m1	FAM
**10.**	*Drd1*	Mm02620146_s1	FAM
**11.**	*Drd2*	Mm00438545_m1	FAM
**12.**	*Drd3*	Mm00432887_m1	FAM
**13.**	*Drd4*	Mm00432893_m1	FAM
**14.**	*Drd5*	Mm04210376_s1	FAM
**15.**	*Kif1a*	Mm00492863_m1	FAM
**16.**	*Kif1b*	Mm00801813_m1	FAM
**17.**	*Kif5a*	Mm00515265_m1	FAM
**18.**	*Kif2c*	Mm00728630_s1	FAM
**19.**	*Dync1h1*	Mm00466548_m1	FAM
**20.**	*Dynll1*	Mm00850282_g1	FAM
**21.**	*Dctn1*	Mm01184845_m1	FAM
**22.**	*Mapt*	Mm00521988_m1	FAM
**23.**	*Map2*	Mm00485231_m1	FAM
**24.**	*Mark2*	Mm01220150_g1	FAM
**25.**	*Tubb3*	Mm00727586_s1	FAM
**26.**	*Tuba1a*	Mm00846967_g1	FAM
**27.**	*Snca*	Mm01188700_m1	FAM
**28.**	*Syn1*	Mm00449772_m1	FAM
**29.**	*Stx1a*	Mm00444008_m1	FAM
**30.**	*Syt1*	Mm00436858_m1	FAM
**31.**	*Syt11*	Mm00444517_m1	FAM
**32.**	*Rab5a*	Mm00727887_s1	FAM
**33.**	*Rab7*	Mm00784318_sH	FAM
**34.**	*Nsf*	Mm00435390_m1	FAM
**35.**	*Dnm1l*	Mm01342903_m1	FAM
**36.**	*Vps35*	Mm00458167_m1	FAM
**37.**	*Sod1*	Mm01344233_g1	FAM
**38.**	*Gpx1*	Mm00656767_g1	FAM
**39.**	*Gsr*	Mm00439154_m1	FAM
**40.**	*Txnrd1*	Mm00443675_m1	FAM
**41.**	*Nos1*	Mm01208059_m1	FAM
**42.**	*Prdx1*	Mm01621996_s1	FAM
**43.**	*Nfe2l2*	Mm00477784_m1	FAM
**44.**	*Keap1*	Mm00497268_m1	FAM
**45.**	*Sigmar1*	Mm01223547_g1	FAM
**46.**	*Park2*	Mm01323528_m1	FAM
**47.**	*Ube2n*	Mm00779119_s1	FAM

48.	*Uba3*	Mm00495866_m1	FAM
49.	*Psmb4*	Mm01263563_m1	FAM
50.	*Psmc3*	Mm00477177_m1	FAM
51.	*Psmd4*	Mm01263490_m1	FAM
52.	*Usp47*	Mm00659716_m1	FAM
53.	*Ubb*	Mm01622233_g1	FAM
54.	*Ifng*	Mm01168134_m1	FAM
55.	*Tgfb1*	Mm01178820_m1	FAM
56.	*Akt1*	Mm01331626_m1	FAM
57.	*Ptgs2*	Mm00478374_m1	FAM
58.	*Traf1*	Mm00493827_m1	FAM
59.	*Cxcl11*	Mm00444662_m1	FAM
60.	*Casp1*	Mm00438023_m1	FAM
61.	*Casp3*	Mm01195085_m1	FAM
62.	*Parp1*	Mm01321084_m1	FAM
63.	*Aifm1*	Mm00442548_m1	FAM
64.	*Bcl2l11*	Mm00437796_m1	FAM
65.	*Map3k5*	Mm00434883_m1	FAM
66.	*Cib1*	Mm00501944_m1	FAM
67.	*Trp53*	Mm01731290_g1	FAM
68.	*Bax*	Mm00432051_m1	FAM
69.	*Fos*	Mm00487425_m1	FAM
70.	*Mapk8*	Mm00489514_m1	FAM
71.	*Lamp2*	Mm00495267_m1	FAM
72.	*Atg16l1*	Mm00513085_m1	FAM
73.	*Atg5*	Mm01187303_m1	FAM
74.	*Tnf*	Mm00443258_m1	FAM
75.	*Ctsb*	Mm01310506_m1	FAM
76.	*Capn1*	Mm00482964_m1	FAM
77.	*Ern2*	Mm00469005_m1	FAM
78.	*Eif2ak3*	Mm00438700_m1	FAM
79.	*Atf6*	Mm01295319_m1	FAM

Table S2. Genes that are not expressed in the substantia nigra in mice

**Gene**	**Protein**
**Monoamines synthesis, degradation**	
Dbh	Dopamine beta-hydroxylase
Pnmt	Phenylethanolamine-N-methyltransferase
**Dopamine transport and reception**	
Drd1	Dopamine receptor D1
Drd3	Dopamine receptor D3
Drd4	Dopamine receptor D4
Drd5	Dopamine receptor D5
**Axonal transport**	
Kif2c	Kinesin family member 2C
**Inflammation and glial activation**	
Ifng	Interferon gamma
Ptgs2	Prostaglandin-endoperoxide synthase 2
Traf1	TNF receptor-associated factor 1
Cxcl11	Chemokine (C-X-C motif) ligand 11
**Cell death**	
Casp1	Caspase 1
Tnf	Tumor necrosis factor
Ern2	Serine/threonine-protein kinase/endoribonuclease IRE2
